# Plant exudates may stabilize or weaken soil depending on species, origin and time

**DOI:** 10.1111/ejss.12487

**Published:** 2017-10-27

**Authors:** M. Naveed, L. K. Brown, A. C. Raffan, T. S. George, A. G. Bengough, T. Roose, I. Sinclair, N. Koebernick, L. Cooper, C. A. Hackett, P. D. Hallett

**Affiliations:** ^1^ School of Biological Sciences University of Aberdeen Aberdeen AB24 3UU UK; ^2^ The James Hutton Institute, Invergowrie Dundee DD2 5DA UK; ^3^ School of Science and Engineering University of Dundee Dundee DD1 4HN UK; ^4^ Faculty of Engineering and Environment University of Southampton Southampton SO17 1BJ UK; ^5^ Biomathematics and Statistics Scotland, Invergowrie Dundee DD2 5DA UK

## Abstract

We hypothesized that plant exudates could either gel or disperse soil depending on their chemical characteristics. Barley (Hordeum vulgare L. cv. Optic) and maize (Zea mays L. cv. Freya) root exudates were collected using an aerated hydroponic method and compared with chia (Salvia hispanica L.) seed exudate, a commonly used root exudate analogue. Sandy loam soil was passed through a 500**‐**μm mesh and treated with each exudate at a concentration of 4.6 mg exudate g^−1^ dry soil. Two sets of soil samples were prepared. One set of treated soil samples was maintained at 4°C to suppress microbial processes. To characterize the effect of decomposition, the second set of samples was incubated at 16°C for 2 weeks at −30 kPa matric potential. Gas chromatography–mass spectrometry (GC–MS) analysis of the exudates showed that barley had the largest organic acid content and chia the largest content of sugars (polysaccharide‐derived or free), and maize was in between barley and chia. Yield stress of amended soil samples was measured by an oscillatory strain sweep test with a cone plate rheometer. When microbial decomposition was suppressed at 4°C, yield stress increased 20‐fold for chia seed exudate and twofold for maize root exudate compared with the control, whereas for barley root exudate decreased to half. The yield stress after 2 weeks of incubation compared with soil with suppressed microbial decomposition increased by 85% for barley root exudate, but for chia and maize it decreased by 87 and 54%, respectively. Barley root exudation might therefore disperse soil and this could facilitate nutrient release. The maize root and chia seed exudates gelled soil, which could create a more stable soil structure around roots or seeds.

**Highlights:**

- Rheological measurements quantified physical behaviour of plant exudates and effect on soil stabilization.
- Barley root exudates dispersed soil, which could release nutrients and carbon.
- Maize root and chia seed exudates had a stabilizing effect on soil.
- Physical engineering of soil in contact with plant roots depends on the nature and origin of exudates.

## Introduction

Soil physical conditions, particularly in the rhizosphere, are continually modified by the release of plant root exudates and microbial metabolites (McCully, 1999; Hinsinger *et al*., 2009). Plants potentially benefit from this modification of the rhizosphere because of improved physical conditions for root penetration, and nutrient and water uptake by the roots (Read *et al*., 2003; Barré & Hallett, 2009). However, the effect of biological exudates on soil physical properties might depend on their physicochemical characteristics (Czarnes *et al*., 2000). The quantity and physicochemical characteristics of root exudates are determined by the plant species, the age of an individual plant and external factors such as biotic and abiotic stresses (e.g. soil structure, presence of microorganisms and nutritional status) (Gransee & Wittenmayer, 2000; Jones *et al*., 2004; Lesuffleur *et al*., 2007). In general, root exudates are composed of an array of compounds such as carbohydrates, amino acids, organic acids, phenolic compounds, fatty acids, sterols, vitamins, enzymes and inorganic molecules. Among these, sugars (often polysaccharide derived), amino acids and organic acids are usually released in the largest quantities (Dakora & Philips, 2002; Carvalhais *et al*., 2011).

There is growing evidence to suggest that certain compounds present in root exudates are involved in engineering the rhizosphere by dispersion and gelling of soil (Tarchitzky & Chen, 2002; Barré & Hallett, 2009; Deng *et al*., 2015), modulation of water and nutrient availabilities (Wang *et al*., 2008; Ahmed *et al*., 2014), and attraction of rhizobacteria (Bais *et al*., 2006). The anions of organic acids in the rhizosphere may be adsorbed by soil mineral particles, thereby increasing the net negative charge of clays that would cause particles to disperse (Shanmuganathan & Oades, 1983). Mucilages and other polysaccharides present in root exudates, which can function as stabilizing materials, might offset this effect (Oades, 1984). The pH of the exudates and thus rhizosphere will also affect soil structural stability if the change in pH results in dissolution or precipitation of stabilizing material such as Al (Yeoh & Oades, 1981) or other polyvalent ions (Oades, 1984).

Root exudates and microbial metabolites can have a marked effect on soil stability and its resistance to disruption from both mechanical and hydraulic stresses. There is considerable evidence suggesting that root exudates improve soil stability. Morel *et al*. (1991) showed that incorporation of maize root exudate in soil resulted in an immediate increase in soil aggregate stability, followed by a decrease over time because of microbial degradation. Traoré *et al*. (2000) also observed a significant increase in the aggregate stability of soil with different substrates such as polygalacturonic acid, modelled soluble exudates and maize root exudate. Czarnes *et al*. (2000) found that adding polygalacturonic acid and xanthan (a bacterial exudate) to soil increased tensile strength and stability against the disruptive effects of wetting and drying cycles. Peng *et al*. (2011) found improved aggregate stability for only certain biological exudates they studied, with large differences between soils containing swelling or non‐swelling clay minerals. They observed improved tensile strength and aggregate stability for soil treated with xanthan, polygalacturonic acid and dextran, but not for lecithin. Compared with the number of studies that reported an increase in soil stability from treatment with plant‐derived biological exudates, those that showed the dispersion of soil with exudates are rare (Tarchitzky & Chen, 2002).

Most research to investigate soil stabilization by biological exudates has measured aggregate stability. Although soil aggregate stability is a relevant test for studying the resistance of soil to erosion, it does not provide a quantitative measure of particle bonding that underpins the formation of soil structure. Fracture tests on dry soil discs (Czarnes *et al*., 2000) or notched bars (Zhang *et al*., 2005) have quantified increased particle bond energy resulting from root exudate compounds, but such dry conditions are rarely met in reality (Hallett *et al*., 2013). Formation of the soil structure occurs when soil is wet and particles are mobile; therefore, rheological measures of wet soil movement under stress are more relevant physically to understanding how mechanical stresses from root growth and exudation affect soil structural development (Barré & Hallett, 2009). The mobility of soil when wet can be described with rheological measurements that provide controlled oscillatory shear stresses to quantify time‐dependent flow under stress (i.e. viscosity and yield stress) (Markgraf *et al*., 2012). Tarchitzky & Chen (2002) found a 10‐fold drop in the viscosity of a liquid suspension of clay minerals when treated with humic acid. The yield stress and viscosity of soil pastes increased markedly if treated with *Capsella bursa‐pastoris* L. Medik (shepherd's purse) seed exudate (Deng *et al*., 2015), the root exudate compound polygalacturonic acid and the fungal exudate scleroglucan (Barré & Hallett, 2009). To our knowledge, no study has characterized the effect of natural root exudates on soil micromechanics, although Read & Gregory (1997) found that maize and lupin root exudates were viscoelastic and far more viscous than water. Such knowledge would greatly improve the understanding of physical formation and stabilization of the rhizosphere.

In the present research we used chia (*Salvia hispanica* L.) seed, barley (*Hordeum vulgare* L. cv. Optic) and maize (*Zea mays* L. cv. Freya) root exudates to test the hypothesis that physical engineering of soil in contact with plants depends on the chemical characteristics of the exudates. Chia seed exudate has been used as a root exudate analogue (Kroener *et al*., 2014), although its behaviour in comparison with natural root exudates is unknown. The first part of this study examined physicochemical characteristics of barley root, maize root and chia seed exudates. In the second part of the study, micromechanical characterization of soil treated with barley root, maize root and chia seed exudates was carried out both before and after decomposition of the exudates in soil. Overall, the research sought to address the following points:

1. How do the chemical characteristics of barley root, maize root and chia seed exudates differ from each other?
2. How do the chemical characteristics of exudates relate to their viscosities?
3. Does yield stress of soil treated with exudates depend on the chemical characteristics of exudates when microbial decomposition is suppressed?
4. How does yield stress of soil treated with exudates change following incubation and associated microbial decomposition of added exudates?

## Materials and methods

### Collection of exudates

#### Extraction of chia seed exudate

Chia seed exudate was extracted following Ahmed *et al*. (2014) by mixing 100 g of distilled water with 10 g of chia seeds with a magnetic stirrer for 2 minutes at 50°C, followed by cooling to room temperature (20°C) and standing for 4 hours. The exudate was separated from the seeds by pushing the mixture repeatedly through a 500‐μm sieve under pressure with a syringe that was cut at the end. This approach harvested the easily extracted seed exudate; the tightly bound exudate remained on the seeds after five repeated extraction attempts. Of 0.13 ± 0.03 (mean ± SE) g g^−1^ total exudate in seeds, only 0.10 ± 0.02 g g^−1^ of seed exudate was harvested; therefore, the extraction efficiency was 77 ± 8%. The extracted exudates were then freeze‐dried. One treatment of chia seed exudate was ball‐milled after freeze‐drying and denoted as BM throughout the paper. This treatment was intended to break up large polymers present in chia seed exudate.

#### Collection of barley and maize root exudates

Barley and maize root exudates were collected by an aerated hydroponic method adopted from Giles *et al*. (2017). Barley (*Hordeum vulgare* L., cv. Optic) and maize (*Zea mays* L. cv. Freya) seeds were surface‐sterilized in sodium hypochlorite solution (2%) for 10 minutes, then rinsed thoroughly in sterile deionized water. Sterilized seeds were pre‐germinated on 1% agar (Sigma‐Aldrich, Gillingham, UK) until the radicals were approximately 1‐cm long (2–3 days post‐germination). After discarding poorly germinated seeds, 180 individuals of barley or maize plants were grown successively in a 60‐l aerated hydroponic tank under the following conditions: illumination, 14 hours and a minimum 200 μmol quanta m^−2^ s^−1^; temperature, day 25°C, night 22°C for maize, and day 18°C, night 14°C for barley growth. Nutrient solutions used in the aerated hydroponic tank were changed every 3 days, beginning with 0.25 strength, followed by 0.5 strength and continuing to full strength until harvest. The full‐strength standard nutrient solution (pH 5.5) contained 3 mm NH_4_Cl, 4 mm Ca(NO_3_)_2_, 4 mm KNO_3_, 1 mm KH_2_PO_4_, 3 mm MgSO_4_ and 0.1 mm Fe‐EDTA with micronutrients (6 μm MnCl_2_, 23 μm H_3_BO_3_, 0.6 μm ZnCl_2_, 1.6 μm CuSO_4_, 1.0 μm Na_2_MoO_4_ and 1.0 μm CoCl_2_). Plants were harvested after 2 weeks of growth. Exudates were collected overnight in 150‐ml pots containing 75 ml distilled water with a set amount of plants per pot (barley × 5 or maize × 3). Plants were removed from the pots the following morning (12‐hour collection period) and the remaining liquid in the collection pots was first frozen at −20°C and then freeze‐dried for collection of the dry barley and maize root exudates. Exudates were collected in distilled water so that root exudates only could be collected after freeze‐drying (in a trial run nutrient solution was used as an exudate collection medium, which produced a large exudate dry weight associated with nutrient salts after freeze‐drying). Harvesting exudates after moving plants to distilled water might induce a change in exudation because of osmotic shock, but we assume here that this is of secondary importance. The average freeze‐dried weight of root exudates collected from individual barley and maize plants was 4.1 ± 0.9 (mean ± SE) and 6.4 ± 1.7 (mean ± SE) mg individual^−1^, respectively.

Freeze‐drying was essential so that the exudates could be concentrated from the dilute collection solutions. The amounts of carbon and nitrogen present in freeze‐dried barley root, maize root and chia seed exudates were measured by CNS elemental analyser (CE Instruments, Wigan, UK). The pH of the exudates at a concentration of 4.6 mg exudate g^−1^ water was measured with a pH meter (Hanna Instruments, Leighton Buzzard, UK).

### Gas chromatography–mass spectrometry (GC–MS) analysis of exudates

Analysis was carried out on an Agilent 5977B GC‐MSD fitted with an HP‐5MS, 5% phenyl, 95% dimethylpolysiloxane, 325°C column (30‐m long, 0.25‐mm internal diameter, 0.25‐μm coating) at an inlet pressure of 68.63 kPa (Agilent, Santa Clara, CA, USA). The freeze‐dried exudates were acid hydrolysed in 0.5 ml of trifluoroacetic acid (TFA) for 1 hour at 70°C; after this time the polymers present had degraded completely. The TFA was removed by drying under a stream of nitrogen and the freeze‐dried exudates were derivatized first by mixing with 0.1 ml of methoxyamine hydrochloride in pyridine (20 mg ml^−1^) in a glass GC sample vial. Vials were incubated at 37°C for 1 hour. After cooling to room temperature, samples were derivatized with 100 μl of N‐methyl‐N‐trimethylsilyltrifluoroacetamide (MSTFA) for 1 hour at 70°C. A 2‐μl subsample was injected directly into the GC–MS for analysis under the following settings: an initial oven temperature of 70°C for 1 minute, a ramp of 5°C minute^−1^ to a temperature of 300°C, which was held for 6 minutes. The total time for the run was 52 minutes. The chemicals relating to each peak detected in the chromatograms were determined with an ‘Agilent MSD Productivity Chemstation for GC and GC–MS Systems Data Analysis Application’ (Agilent) by matching to the NIST (National Institute of Standards and Technology) 11 database. Results are presented as the original chemicals present in the samples, with removal of the derivatization groups where possible. Chia seed exudate was analysed in triplicate to confirm the reproducibility of the results, and then barley and maize root exudates were analysed only once.

### Viscosity measurement of the exudate solutions

Freeze‐dried barley root, maize root and chia seed exudates (freeze‐dried and freeze‐dried, ball‐milled) were mixed into distilled water to achieve a concentration of 4.6 mg g^−1^. This is a realistic exudate concentration in the rhizosphere, as shown in Zickenrott *et al*. (2016). The viscosity of these exudate solutions was then measured with a Discovery Hybrid Rheometer HR‐3 (TA Instruments, New Castle, DE, USA) equipped with a cone‐plate geometry (60 mm diameter, 1° angle). Stress sweep tests were carried out under the following conditions: a gap of 500 μm, normal force initially at 0 N and restricted to <0.1 N during testing, five measurement points for every order of magnitude of applied stress, test temperature 20°C and test duration 15 minutes. After placing enough exudate solution (1.5 ml) between the plates, the viscosity of the solution was measured in triplicate by applying a sequence of constant stress values to the samples and measuring the corresponding shear rate. Two viscosities were derived from the apparent viscosity curves (i.e. zero‐shear viscosity and infinite‐shear viscosity). Root exudates are shear‐thinning materials, which means that as an increasing shear stress is applied, they become progressively weaker. Below the yield stress, shear stress has no effect on viscosity, and so the material is at the zero‐shear viscosity. The large shear‐rate‐limiting value of viscosity is called infinite shear viscosity. This is usually associated with shear thinning when liquids flow more easily under stress, and further increases in stress have little effect on viscosity.

### Selection and preparation of soil

A Eutric Cambisol under barley production was collected from 0–100‐mm depth in Bullion Field at the James Hutton Institute (JHI), Dundee (56°27′ 39″ N, 3°04′11″ W). The soil has a sandy loam texture (clay = 16%, silt = 24%, sand = 60%) determined by the combination of wet sieving and hydrometer methods. It had 22.5 g kg^−1^ total carbon, 1.6 g kg^−1^ total nitrogen and soil pH in CaCl_2_ of 5.48. The soil was partially air‐dried to 150 g kg^−1^ and then passed through a 500‐μm sieve to decrease particle interlocking in the rheological tests. The sieved soil was then mixed with either distilled water (unamended) or each of the exudates: barley root, maize root, chia seed and chia seed exudates after ball‐milling at a concentration of 4.6 mg exudate g^−1^ dry soil.

Two separate experiments were carried out: experiment 1 explored the effect of exudates on the micromechanics of soil and experiment 2 explored the effect of decomposition of exudates on the micromechanics of soil. For experiment 1, six to seven subsamples for each treatment were prepared with increasing water contents from 350 to 550 g kg^−1^. All the subsamples were incubated at 4°C for 24 hours in sealed plastic bags to homogenize. Oscillatory strain sweep tests were then performed on each subsample with the same rheometer and plate set‐up used to characterize exudate viscosity. For experiment 2, six subsamples for each treatment were prepared and incubated at −30 kPa matric potential at 16°C for 2 weeks in Kilner Jars for decomposition. After incubation, individual subsamples were adjusted to increasing water contents from 350 to 450 g kg^−1^, and were again incubated at 4°C for 24 hours in sealed plastic bags to homogenize. Oscillatory strain sweep tests were then performed with the rheometer mentioned above on each subsample by placing the same amount of soil paste (7 g) under the cone‐plate for all the treatments.

### Oscillatory strain sweep tests

**Figure 1:**
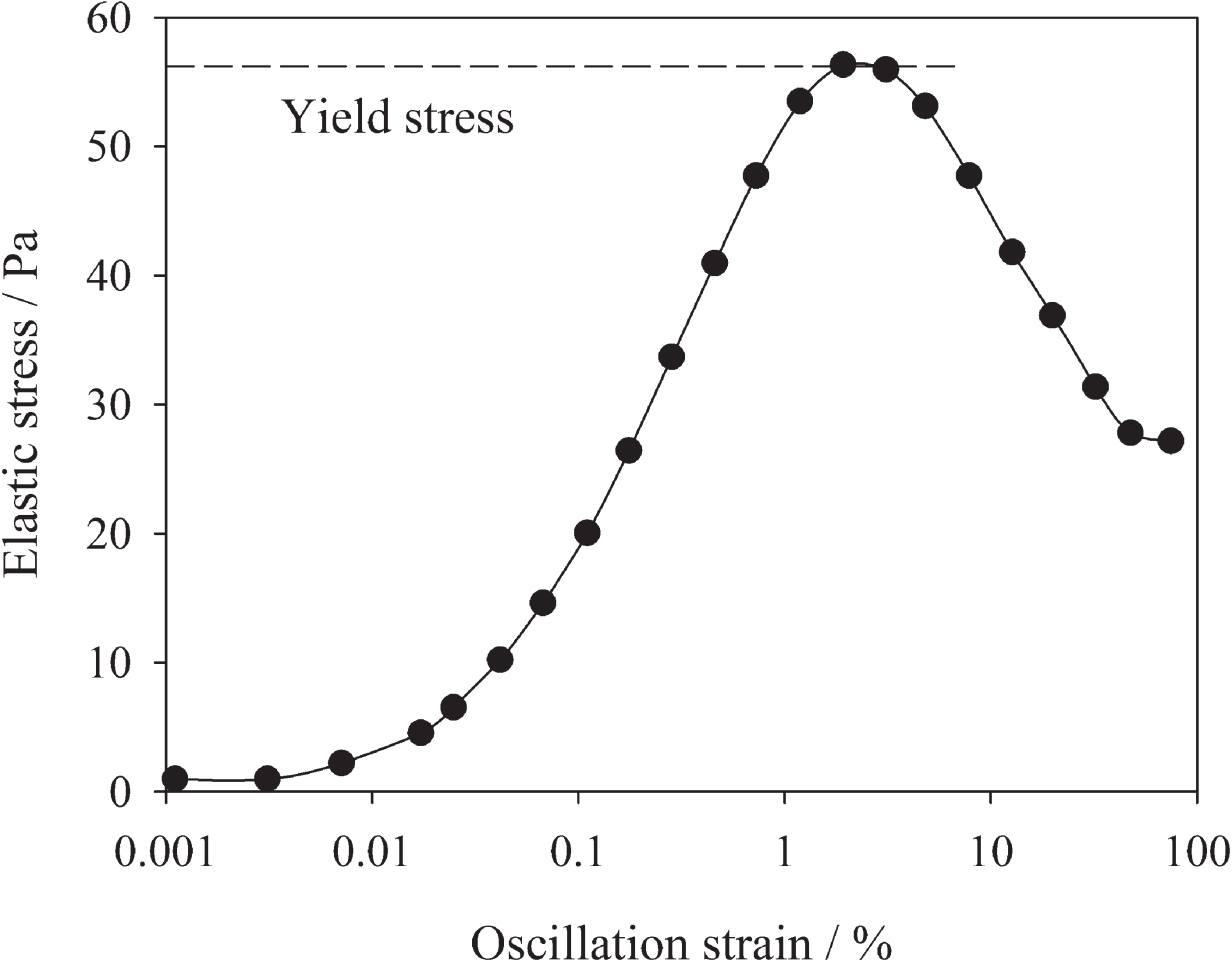
Analysis of an oscillatory strain sweep test; elastic stress was plotted as a function of oscillation strain. The dashed line shows the calculation of yield stress from the data.

Pre‐settings of the oscillatory strain sweep tests used are given in Table 1. An oscillatory sweep test stresses and then relaxes a specimen under shear; at each step an increased shear strain is applied. The elastic stress was plotted as a function of oscillation strain in Figure 1. The peak elastic stress was denoted as yield stress and corresponding strain was denoted as yield strain as suggested by Walls *et al*. (2003). The yield stress is the onset of soil structural collapse, which generally lies between the linear viscoelastic range and flow point. We reported yield stress instead of yield viscosity; they are directly correlated at an angular frequency of 1 Hz, which was used here. Furthermore, we preferred yield stress to viscosity for soil pastes because it has a unique maximum point in elastic stress when plotted against oscillation strain where elastic stress starts to decrease with further increase in oscillation strain (Figure 1).

**Table 1:** Pre‐settings of oscillatory strain sweep tests on exudate‐amended soil

Property	Symbol and value
Plate gap	*d* = 2 mm
Cone‐plate radius	*R* = 30 mm
Cone‐plate angle	*θ* = 1°
Oscillation strain	*γ* = 0.001–1000%
Measured points for each test	30
Duration	Approximately 15 minutes

### Statistical analysis

Soil yield stress was modelled using general linear regression analysis with logarithmically (base 10) transformed soil yield stress as the response variate, water content as the explanatory variate, and exudates and decomposition as factors (the regression intercept constants for yield stress were evaluated at 350 g kg^−1^ water content). The same regression analysis was then repeated, but excluding the chia seed mucilage treatment because it was markedly different from the maize and barley exudate treatments. Summary tables for the accumulated analysis of variance (anova) obtained with the general linear regression analysis are reported in the results.

To facilitate interpretation of the regression coefficients with respect to decomposition, a new factor was formed with distinct levels for each combination of exudate and decomposition and a further regression analysis was performed on water content and this factor to estimate the intercept and slope for each treatment combination. Differences between intercept values for individual pairs of treatments of interest were determined using t‐tests of pairwise differences. Statistical analyses were carried out with Genstat version 18 (VSN International Limited, Oxford, UK).

## Results

### Chemical characterization of the exudates

Total carbon contents of freeze‐dried barley root, maize root and chia seed exudates were 149, 166 and 407 g kg^−1^, respectively. Total nitrogen contents of freeze‐dried barley root, maize root and chia seed exudates were 62, 33 and 11 g kg^−1^, respectively. This resulted in C/N ratios of the exudates of 2.4 for barley root, 5.1 for maize root and 37.0 for chia seed. The pH of the aqueous exudate solutions at 4.6 mg g^−1^ concentration was 8.9 for barley root, 9.35 for maize root and 6.7 for chia seed.

**Figure 2:**
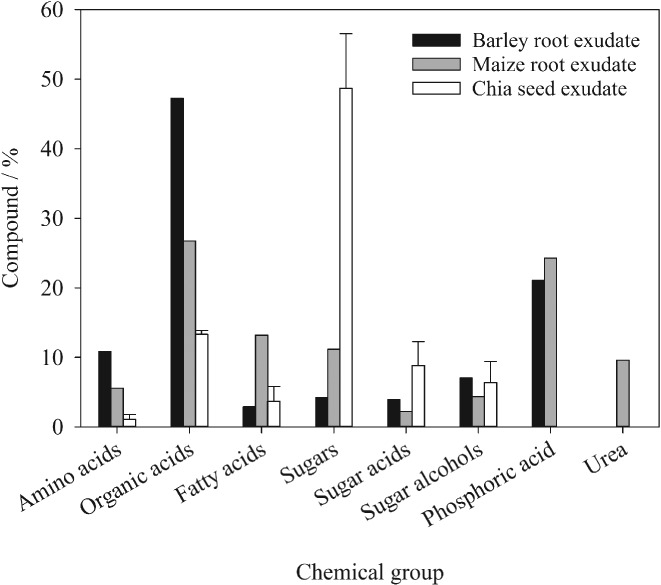
Chemical characterization of the barley root, maize root and chia seed exudates by gas chromatography–mass spectrometry (GC–MS). Error bars are the standard errors (SEs). Note that sugars listed are probably largely polysaccharide derived, following acid hydrolysis of the exudates.

The major chemical groups identified in barley root, maize root and chia seed exudates by GC–MS are shown in Figure 2. Barley root exudate had 10.8% amino acids, 47.2% organic acids, 2.8% fatty acids, 15% sugars, sugar acids and sugar alcohols (note that sugars are probably largely polysaccharide derived following acid hydrolysis) and 15% phosphoric acid. Maize root exudate had 5.7% amino acids, 27.8% organic acids, 13% fatty acids, 17.8% sugars, sugar acids and sugar alcohols, 24% phosphoric acid and 9.6% urea. Chia seed exudate had 1.1% amino acids, 13.3% organic acids, 2% fatty acids and 64% sugars, sugar acids and sugar alcohols. Thus organic acids and sugars were the major compounds in all three exudates. Organic acids were present in the largest amount in barley root followed by maize root and chia seed exudates. It was the reverse for sugars (i.e. chia seed > maize root > barley root).

The number of different chemical compounds identified by GC–MS was 50 for barley root, 113 for maize root and 63 for chia seed exudates. Barley root exudates comprised seven amino acids, eight organic acids, 21 sugars and sugar acids, five fatty acids and phosphoric acid. Maize root exudates comprised eight amino acids, 18 organic acids, 52 sugars and sugar acids, 11 fatty acids and phosphoric acid together with urea. Chia seed exudates comprised three amino acids, eight organic acids, 29 sugars and sugar acids and 16 fatty acids. Major chemical compounds and their relative amounts present in barley root, maize root and chia seed exudates are listed in Table 2. Although there was more sugar in chia seed exudate, the diversity in sugar compounds was greater for maize root exudate. Similarly, although more organic acid was observed in barley root exudates, the diversity in organic acids was again more in maize root exudates.

**Table 2:** Major chemical compounds as a percentage of total dry mass by weight in barley root, maize root and chia seed exudates

Chemical group	Compound / g 100 g^−1^					
	Barley		Maize		Chia	
Amino acid	Glycine	4.89	Valine	2.90	Threonine	1.27
	Alanine	4.61	Alanine	1.14	Ketobutyric acid	0.52
	Valine	0.51	Isoleucine	0.87		
			Glycine	0.67		
Organic acid	Butanoic acid	39.03	Butanoic acid	17.94	Oxalic acid	3.27
	Acetoacetic acid	5.55	Acetoacetic acid	2.02	Pentenimidic acid	3.05
	Succinic acid	1.13	Succinic acid	0.94	Allonic acid	1.44
			Lactic acid	0.91	Sebacic acid	1.36
			Malonic acid	0.68	Succinic acid	0.78
					Arachidonic acid	0.69
Fatty acid	Palmitic acid	2.05	Adipic acid	5.02	Palmitic acid	4.65
	Stearic acid	0.55	Palmitoleic acid	3.27	Adipic acid	4.20
			Oleic acid	2.15		
			Stearic acid	1.22		
			Linoleic acid	0.56		
Sugar	Gulose	2.18	Galactose	2.22	Ribose	21.03
	Galactose	0.59	Talose	1.32	Pentose	10.47
			Psicose	1.22	Ribitol	9.64
			Sorbose	1.15	Xylose	7.61
			Rhamnose	1.06	Arabinose	2.03
			Maltose	0.71	Galactofuranose	1.51
			Ribose	0.66	Mannose	1.19
			Fructose	0.58		
Sugar acid	Ribonic acid	2.30	Threonic acid	1.22	d‐Arabinonic acid	1.46
	Gluconic acid	1.18	Gluconic acid	0.84	Glucaric acid	0.88
	Threonic acid	0.88			Galactonic acid	0.74
					Glucuronic acid	0.70
					d‐Galacturonic acid	0.69
					Gluconic acid	0.67
Sugar alcohol	Myo‐inositol	6.86	Myo‐inositol	3.66	Myo‐inositol	8.20
			Xylitol	0.58	Threitol	1.36
Others	Phosphoric acid	21.0	Phosphoric acid	24.29		
			Urea	9.64		

Compounds weighing >0.5 g 100 g^−1^ only are given here.

Note that sugars listed are probably largely polysaccharide derived, following acid hydrolysis of the exudates.

### Viscosity of the exudate solutions

**Figure 3:**
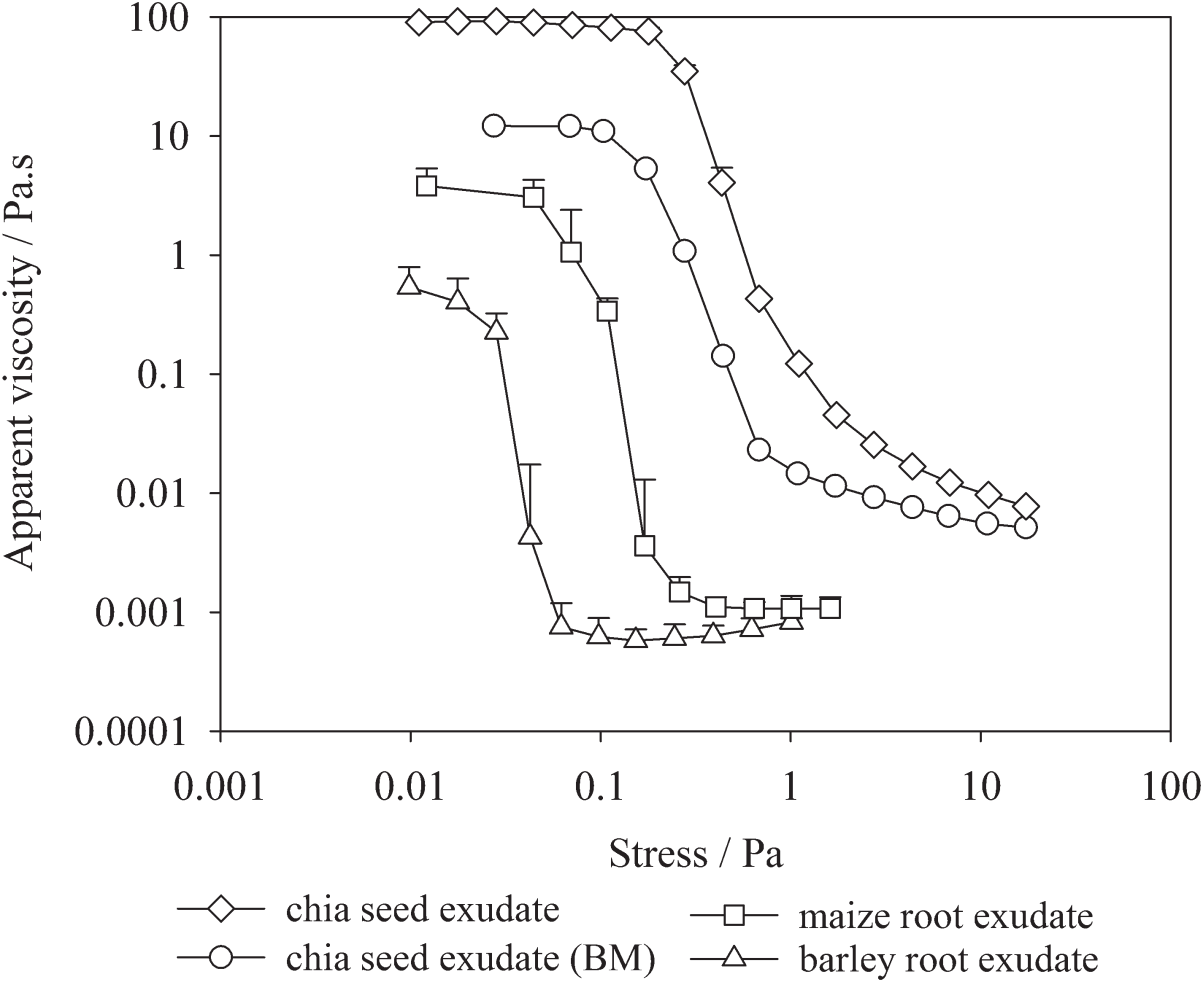
Apparent viscosity (mean + standard error [SE]) plotted as a function of applied stress for chia seed, chia seed after ball milling (BM), barley root and maize root exudates at a concentration of 4.6 mg exudate g^−1^ water.

The apparent viscosity of barley root, maize root, chia seed and chia seed exudates after ball milling (BM) at 4.6 mg g^−1^ concentration as a function of applied stress is shown in Figure 3. The small value of stress is associated with zero‐shear viscosity, which for exudates was 95.1 Pa s for chia seed, 12.1 Pa s for chia seed (BM), 2.4 Pa s for maize root and 0.4 Pa s for barley root. Infinite‐shear viscosity for chia seed, chia seed (BM), maize root and barley root exudates was 9, 8, 1.4 and 0.6 mPa s, respectively. Exudates were composed mainly of sugars and organic acids, and both zero‐ and infinite‐shear viscosities of the exudates were positively correlated with the amount of sugars and negatively correlated with the amount of organic acids. More sugar was associated with the large viscosity of the chia seed, followed by maize and then barley root exudate (Figure 3).

### Rheological characterization of exudate‐treated soil before decomposition

**Figure 4:**
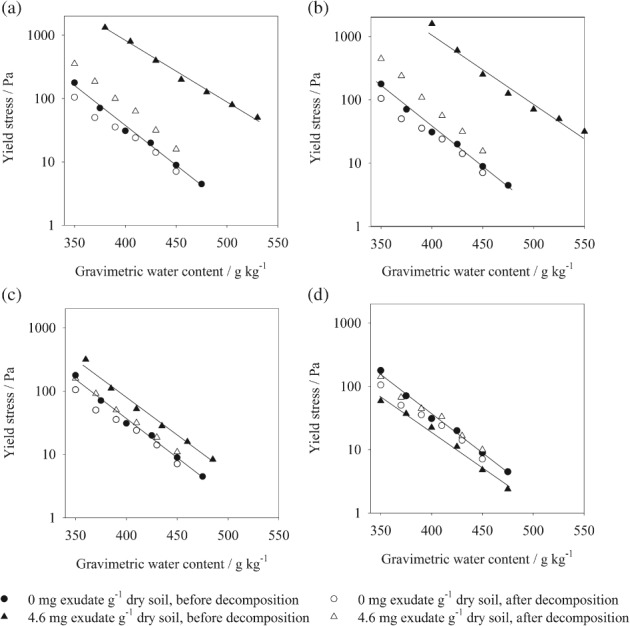
Yield stress of soil treated with (a) chia seed, (b) chia seed after ball‐milling (BM), (c) maize root and (d) barley root exudates, together with unamended soil both before and after decomposition plotted as a function of water content. Fitted lines are shown for the data measured before decomposition only.

Yield stress decreased significantly with increasing water content, regardless of exudate amendment (Figure 4, Tables 3 4). Yield stresses were significantly greater for soil treated with chia seed and maize root exudates and smaller for barley root exudate than for unamended soil over a range of water contents (Figure 4, Tables 3 4). In general linear regression analysis we evaluated the intercept yield stress at 350 g kg^−1^ water content, the minimum water content at which it was practical to perform an oscillation sweep test. Soil treated with chia seed (*P* < 0.01) and maize root (*P* < 0.01) exudates had significantly larger intercept yield stresses than the unamended soil (Table 5). Soil treated with chia seed exudates had the greatest effect; the intercept yield stress increased 20‐fold compared with the unamended soil. The intercept yield stress for soil treated with maize root exudate was twice that of unamended soil (Table 5). Our most surprising finding was the smaller yield stresses observed for soil treated with barley root exudate than for the unamended soil over a range of water contents (Figure 4). The intercept yield stress for soil treated with barley root exudate was significantly smaller (*P* < 0.01) than that for the unamended soil. The intercept yield stress for soil treated with barley root exudate was almost half that of unamended soil (Table 5).

**Table 3:** Accumulated analysis of variance obtained by general linear regression analysis for logarithmically (base 10) transformed soil yield stress as response variate, water content as explanatory variate, and exudates and decomposition as two factors

Source	d.f.	SS	MS	Var.	*P*
Water content	1	4.12	4.12	1081	<0.001
Decomposition	1	2.46	2.46	644	<0.001
Water content·decomposition	1	0.18	0.18	48	<0.001
Exudates	4	10.89	2.72	715	<0.001
Water content·exudates	4	0.83	0.21	54.2	<0.001
Decomposition·exudates	4	3.76	0.94	246	<0.001
Water content·decomposition·exudates	4	0.11	0.03	7.2	<0.001
Residual	42	0.16	0.04		
Total	61	22.51	0.37		

The intercept was fixed at 350 g kg^−1^ water content, the minimum water content where the oscillatory strain sweep test was carried out for a given soil.

d.f., degrees of freedom; SS, sum of squares; MS, mean squares; Var., variance ratio; *P* = *F*‐probability.

**Table 4:** Accumulated analysis of variance as for Table 3 excluding chia seed exudate treatments

Source	d.f.	SS	MS	Var.	*P*
Water content	1	7.12	7.12	2287	<0.001
Decomposition	1	0.014	0.014	4.46	0.045
Water content·decomposition	1	0.005	0.005	1.61	0.22
Exudates	2	0.73	0.36	118	<0.001
Water content·exudates	2	0.007	0.004	1.13	0.34
Decomposition·exudates	2	0.52	0.26	83.4	<0.001
Water content·decomposition·exudates	2	0.003	0.001	0.47	0.63
Residual	24	0.075	0.003		
Total	35	8.47	0.242		

d.f., degrees of freedom; SS, sum of squares; MS, mean squares; Var., variance ratio; *P* = *F*‐probability.

**Table 5:** Intercept (c) and slope (m) were evaluated by general linear regression analysis using logarithmically (base 10) transformed soil yield stress as response variate, water content as explanatory variate, and exudates and decomposition together as one factor

Exudate treatment	*m*	*c*
Before decomposition		
0 mg g^−1^	−0.12 ± 0.009	2.19 ± 0.045
Barley root exudate, 4.6 mg g^−1^	−0.11 ± 0.009	1.84 ± 0.045
Maize root exudate, 4.6 mg g^−1^	−0.12 ± 0.009	2.52 ± 0.050
Chia seed exudate, 4.6 mg g^−1^	−0.10 ± 0.008	3.39 ± 0.054
Chia seed exudate (BM), 4.6 mg g^−1^	−0.11 ± 0.009	3.61 ± 0.063
After decomposition		
0 mg g^−1^	−0.11 ± 0.010	1.99 ± 0.045
Barley root exudate, 4.6 mg g^−1^	−0.11 ± 0.007	2.11 ± 0.045
Maize root exudate, 4.6 mg g^−1^	−0.12 ± 0.010	2.19 ± 0.045
Chia seed exudate, 4.6 mg g^−1^	−0.13 ± 0.010	2.55 ± 0.045
Chia seed exudate (BM), 4.6 mg g^−1^	−0.15 ± 0.010	2.65 ± 0.045

Intercepts were evaluated at 350 g kg^−1^ gravimetric water content, the minimum water content where the oscillatory strain sweep test was carried out for a given soil.

**Figure 5:**
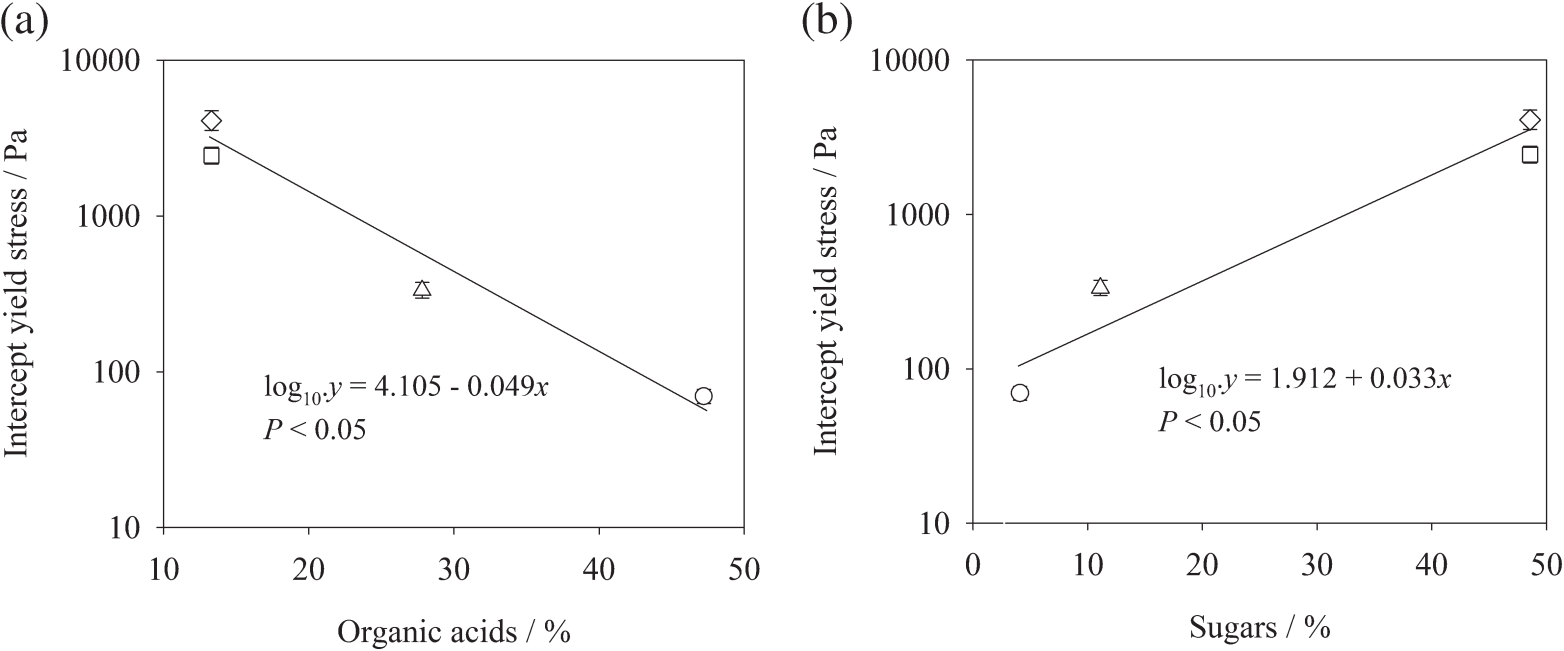
Intercept yield stress (mean ± standard error [SE]) obtained from general linear regression analysis at 350 g kg^−1^ water content (see Table 5) of soil treated with barley root (circle), maize root (triangle), chia seed (square) and chia seed exudates after ball‐milling (diamond) at 4.6 mg exudate g^−1^ dry soil plotted as a function of (a) amount of organic acids and (b) amount of sugars (polysaccharide derived or free) present in the exudates.

The slope of the linear model for chia seed exudate was significantly less than for the unamended soil (*P* < 0.01). The slopes of the linear model for barley and maize root exudates were not significantly different from that for unamended soil (Table 4 5). The yield stress of the samples treated with exudates was strongly related to the chemical characteristics of the exudates. There was a significant negative correlation between the amount of organic acids in the exudates and intercept yield stress of the exudate‐treated soil (Figure 5a), but a positive correlation with the sugars in the exudates (Figure 5b). Exudates were composed mainly of organic acids and sugars, and so the yield stress of the exudate‐treated soils and the quantity of sugars in exudates appear to be positively correlated (Figure 5b).

### Rheological characterization of exudate‐treated soil after decomposition

The aim was to test the role of microbial metabolites generated from the decomposition of plant exudates in the gelling or dispersion of the soil. After the 2‐week incubation period used, almost all of the added exudates were decomposed (data not shown). The yield stress of soil treated with exudates after decomposition showed some interesting differences between the exudates (Figure 4). A significant effect of decomposition on soil yield stress was observed for all exudate treatments (Table 3). Significantly smaller intercept yield stresses (*P* < 0.01) were observed after decomposition than before decomposition for soil treated with both chia seed and maize root exudates (Table 5). The intercept yield stress decreased by 87% for soil treated with chia seed exudate and 54% for maize root exudate after decomposition (Table 5). Barley root exudate initially decreased the yield stress, thereby weakening the soil, but after microbial decomposition, the yield stress increased to approach that of the untreated control (Figure 4). The intercept yield stress of soil for barley root exudate treatment was significantly increased (Tables 3 4). The intercept yield stress increased by 85% for soil treated with barley root exudate after decomposition compared with that before decomposition (Table 5). After decomposition, the slopes of the linear model for chia seed exudates increased significantly (*P* < 0.01), whereas slopes for barley and maize root exudates did not differ significantly from those before decomposition (Table 5).

## Discussion

Various studies have reported a range of carbon (C) and nitrogen (N) contents in root exudates from the same crop. For example, Morel *et al*. (1986) measured 369 g kg^−1^ C and 35 g kg^−1^ N in maize root exudates. They collected maize root exudates from aerated hydroponic growth followed by suction of exudates directly off roots. Pojasok & Kay (1990) measured 110–460 g kg^−1^ C in maize root exudates. The collection of exudate involved growing plants in sand followed by leaching. Traoré *et al*. (2000) found 345 g kg^−1^ C and 4.3 g kg^−1^ N in maize root exudates collected from plants grown in the field. The differences in C and N in maize root exudates between our study and earlier research could be a result of different growth conditions and methods of collecting root exudates. The pH of the maize root exudate solution was similar to that of Pojasok & Kay (1990). We also found that the organic acids and sugar compounds we recorded were similar to those of other studies, for example in root exudates from rice (Bacilio‐Jiménez *et al*., 2003), maize (Carvalhais *et al*., 2011) and lettuce (Neumann *et al*., 2014). However, it is difficult to compare these chemical compounds quantitatively between different studies because exudates depend on many factors, such as method of collection, plant species, the age of an individual plant and external factors such as biotic and abiotic stresses, including soil structure, presence of microorganisms and nutrient status. The proportion of phosphoric acid in the root exudates was larger than expected. We ran a set of blanks again to check the source of phosphoric acid, but it was not detectable. This means that the source of phosphoric acid could be the cells that sloughed off at the root caps into the water during the collection period.

The physical characteristics of some of the exudates were in accord, like the chemical characteristics, with the limited body of research that provides comparable data. The viscosity of *Capsella* seed exudate measured by Deng *et al*. (2013) agreed well with the zero‐shear viscosity of chia seed exudate at similar concentrations in the present study. Bais *et al*. (2005) reported that zero‐shear and infinite‐shear viscosities for scleroglucan (a fungal exudate) were 10 times greater than for chia seed exudate at similar concentrations. Large standard errors observed for barley and maize root exudates (Figure 3) were probably because of (i) a sharp decrease in viscosity with increasing shear stress near the inflection point and (ii) water‐insoluble material present in root exudates. The source of this water‐insoluble material could be the cells that sloughed off the root caps into the water during exudate collection. The infinite‐shear viscosities of maize root exudate were comparable to those of Read & Gregory (1997); they measured viscosity from flow characteristics through a capillary tube for exudates collected directly from root tips. The variation in viscosities between different exudates could be attributed to polysaccharide sugars in the exudates (i.e. more polysaccharide sugars in the exudates resulted in greater viscosities) (Read & Gregory, 1997). Greater zero‐shear and infinite‐shear viscosities for chia seed exudate are consistent with the presence of large amounts of sugars. The viscosity of the barley and maize root exudates represented relatively weak resistance against shear and would present only slight differences in capillary behaviour. Thus resistance to flow in soil pores because of increased viscosity of the soil solution might be minimal, although particles might gel to surrounding soil.

Both chia seed and maize root exudates gelled soil particles, resulting in stabilization that might increase soil aggregation. The largest increase in yield stress was for soil treated with chia seed exudates compared with the unamended soil and was linked to the largest amount of sugars or polysaccharides in this exudate. This is consistent with the binding of mineral soil particles by polysaccharide sugars, which stabilizes the soil (Oades, 1984). The behaviour of soil treated with chia seed and maize root exudates in this study was consistent with the root exudate compound polygalacturonic acid (PGA), which also increased the viscosity of clays considerably (Tarchitzky & Chen, 2002; Barré & Hallett, 2009). An increase in the viscosity of soil treated with *Capsella* sp. seed exudate, similar to that of chia seed, was also reported by Deng *et al*. (2015). The smaller yield stresses for soil treated with barley root exudate than for unamended soil might arise from changes in interparticle bonding. The anions of organic acids, present in large amounts in barley root exudate, might be adsorbed on the mineral soil particles, which might in turn increase the net negative charge of clays and result in greater clay dispersibility (Shanmuganathan & Oades, 1983). This could potentially increase the release of nutrients and carbon from soil through the exposure of new particle surfaces, but it is in contrast to the widely measured increase in physical stability of rhizosphere soil (Morel *et al*., 1991; Traoré *et al*., 2000). In short, the relative amounts of sugars and organic acids in the exudates were consistent with changes in the mechanical stabilization of exudate‐treated soil. For example, organic acids dominated the barley root exudate and probably resulted in dispersion of the soil treated with this exudate. Similarly, sugars that include polysaccharides dominated the chia seed exudate and presumably resulted in gelling of soil particles.

The rhizosphere contains abundant microbial communities that use root exudates and rhizodeposits as substrate (Bais *et al*., 2006; Nihorimbere *et al*., 2011). These organisms produce a range of microbial metabolites that provide a range of ecological functions, including phytostimulation and a defence mechanism by serving as biocontrols (Badri & Vivanco, 2009). On decomposition, the yield stresses of soil treated with both chia seed and maize root exudates decreased significantly compared with those before decomposition. This might result from microbial metabolites that decrease gelling ability compared with both maize root and chia seed exudates. Furthermore, Morel *et al*. (1991) also showed that incorporation of maize root exudate into soil resulted in an immediate increase in soil aggregate stability; thereafter the percentage of water‐stable aggregates decreased rapidly with microbial degradation. The barley root exudates initially decreased yield stress of the soil, which was reversed with microbial decomposition. This suggests that the immediate effect of barley root exudate on the soil was particle movement that could release nutrients, whereas after decomposition a more stable physical structure in the rhizosphere results. Dorioz *et al*. (1993) provided visual evidence from scanning electron microscopy of soil dispersion followed by aggregation in the rhizosphere. In Figure 4b of Dorioz *et al*. (1993), clay plates are orientated parallel to the root surface and the onset of aggregation is evident slightly further away.

Our physical quantification of the micromechanics of soil has resolved some of the underlying physical processes involved in rhizosphere formation and soil aggregation. Exudates rich in organic acids, such as the barley root exudate in this research, have a net dispersing effect on soil in contact with roots. However, with microbial decomposition this dispersing effect decreased and the soil became more stable. The exudates rich in sugars, maize root and chia seed exudates in this study, have a net stabilizing effect on soil in contact with roots from the onset. There is considerable scope to extend this research to explore whether dispersion from particular root exudates might release physically protected carbon, causing a priming effect. Keiluweit *et al*. (2015) found that oxalic acid in root exudates liberates physically protected organic matter in soil, but they did not measure the physical mechanism. Moreover, root exudates from different genotypes of the same crop are known to cause large differences in microbial community structure and function of the rhizosphere (Mwafulirwa *et al*., 2016; Pieterse *et al*., 2016). Perhaps there is an opportunity to breed crops that could potentially manipulate the rhizosphere physically to improve resource availability and resist abiotic stresses (White *et al*. 2013).

## Conclusions

The mechanical tests reported here show the effects of species and decomposition on the stability of exudate‐amended soil. Barley root exudate weakened soil, followed by strengthening after biological decomposition. The initial weakening of soil by barley root exudate might help in releasing previously inaccessible nutrients from soil by dispersion. Maize root and chia seed exudates, on the other hand, strengthen soil from the onset, with biological decomposition decreasing strength. This strengthening of soil by maize root and chia seed exudates could increase the stable soil structure commonly observed near roots.

The chemical characteristics of barley root, maize root and chia seed exudates analysed by GC–MS were quite different from each other; barley had the largest amount of organic acids, but the least amount of (polysaccharide‐derived or free) sugars. This was reflected in the yield stress of exudate‐amended soil, which was negatively correlated with the amount of organic acids and positively correlated with the amount of (polysaccharide‐derived or free) sugars in the exudates. Chia seed exudate has limitations as a model root exudate because of its different chemical characteristics and exaggerated effect on soil physical behaviour compared with that of barley and maize root exudates.

The use of root exudates collected by the aerated hydroponic method in this study is a considerable improvement on the use of model root exudate compounds in previous research. We appreciate that this approach might produce root exudates with a different composition from what would be produced in a soil environment. It is almost impossible to collect exudates in the required amount for mechanical testing from plants grown in soil. Our next step is to develop a micromechanical indentation probe to measure soil strength at the root–soil interface so that the effect of soil conditions and different plants can be explored under more realistic conditions.
